# Unveiling the Truth: Diagnosing Bacterial Meningitis Through Repeat Lumbar Punctures

**DOI:** 10.7759/cureus.40811

**Published:** 2023-06-22

**Authors:** Abeer Qasim, Minu C Abraham, Nismat Javed, Patrik Schmidt, Joshua Davidson

**Affiliations:** 1 Internal Medicine, BronxCare Health System, New York, USA; 2 Internal Medicine, BronxCare Health System, Icahn School of Medicine at Mount Sinai, New York, USA; 3 Pulmonary and Critical Care Medicine, BronxCare Health System, New York, USA

**Keywords:** decoding bacterial meningitis -the crucial role of lumbar puncture in diagnosis, lumbar puncture -a vital diagnostic tool in bacterial meningitis, bacterial cns infection, bacterial cerebrospinal meningitis, bacterial meningitis and the need for repeated lumbar puncture

## Abstract

Bacterial meningitis is a cause of global concern given its associated high rates of mortality and complications. Timely diagnosis and management are crucial in improving outcomes in patients. Lumbar puncture and radiological investigations form the crux of diagnosis. However, the clinical course becomes complicated if lumbar puncture results are unrevealing and equivocal for bacterial meningitis. We present a case of a 60-year-old female who was diagnosed with bacterial meningitis on repeated lumbar puncture. Clinical vigilance and a high degree of suspicion is needed to ensure that patients with bacterial meningitis are diagnosed and managed appropriately, especially in cases with inconclusive lumbar puncture or radiological investigations.

## Introduction

Meningitis, a serious infection of the meninges, can be caused by bacteria, viruses, parasites, and fungi. The incidence of cases has increased from 2.50 million (95% UI 2.19-2.91) in 1990 to about 2.82 million in 2016 [1]. Bacterial meningitis is primarily responsible for a major part of the burden of the disease, with cases exceeding 16 million worldwide [2]. Clinical features include neck stiffness, fever, and altered consciousness in less than 50% of patients with acute bacterial meningitis [3]. The gold standard for diagnosing meningitis is the examination of the cerebrospinal fluid (CSF) and lumbar puncture (LP) [3]. Typically results reveal high protein and low glucose. Cerebrospinal fluid lactate, however, is important in differentiation as it is unaffected by serum concentration [3]. However, there is limited evidence about the repetition of LP. Here, we present a 60-year-old female with a history of headaches who had initially negative Lumbar punctures, and was later diagnosed with meningitis after a second lumbar puncture was performed on clinical deterioration.

## Case presentation

**Figure 1:**
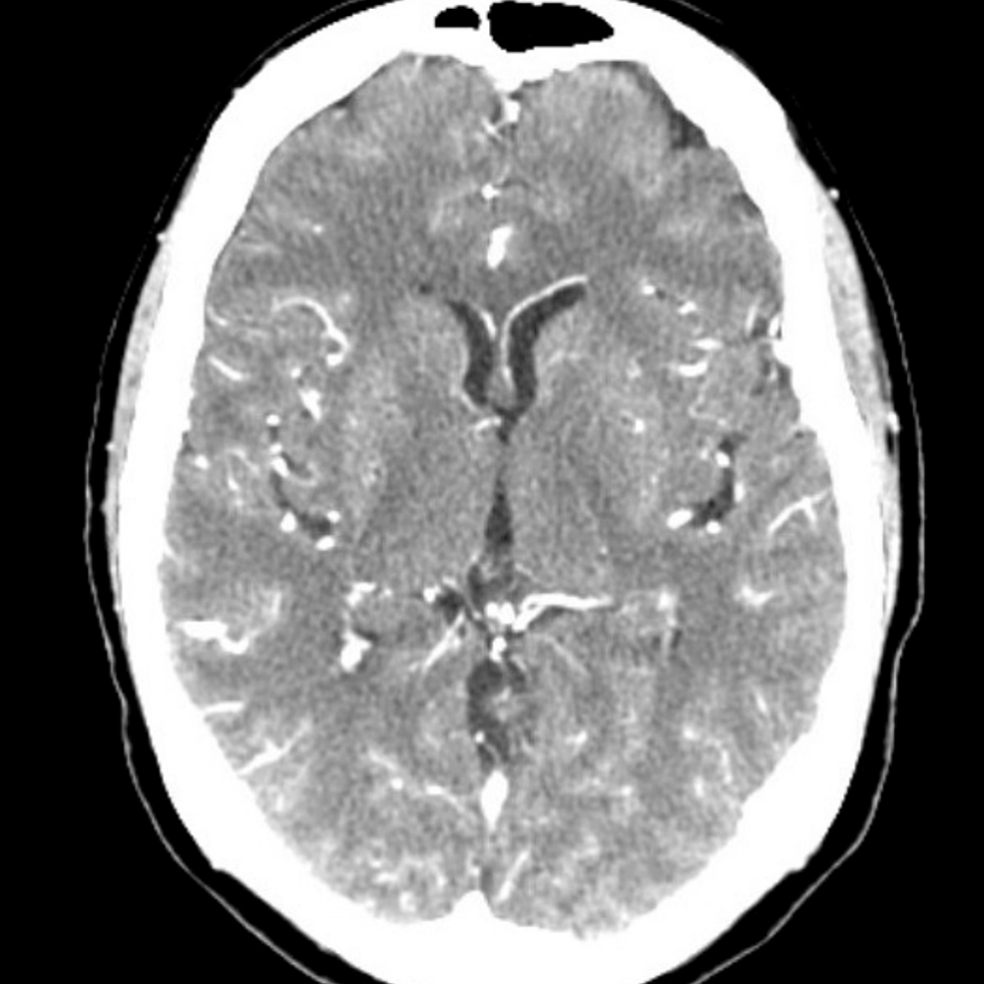
CT angiography head with and without contrast: no acute findings.

**Figure 2:**
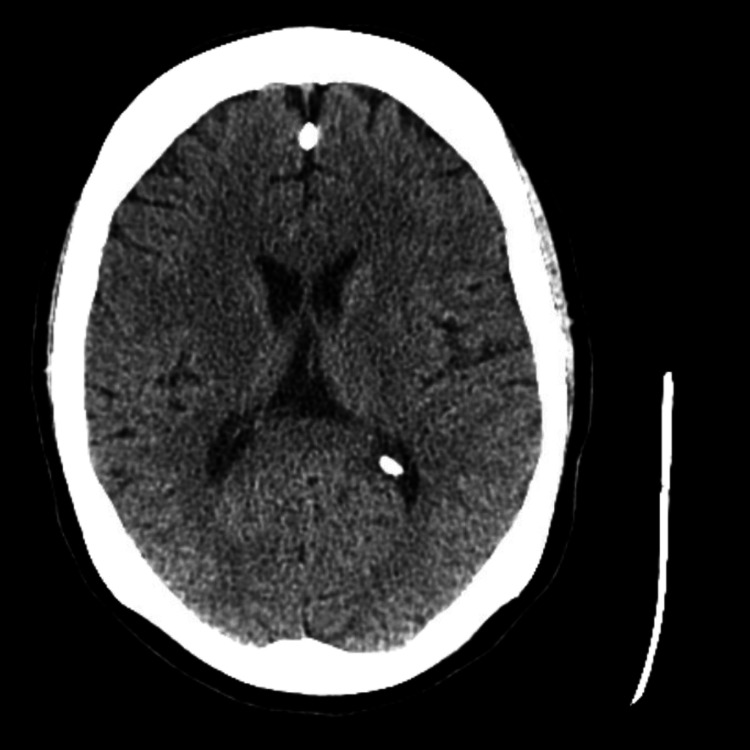
CT brain without contrast: no acute findings in the head/brain.

Our patient is a 60-year-old female with a past medical history significant for type II diabetes, dyslipidemia, major depressive disorder, osteoarthritis, rheumatoid arthritis, and chronic low back pain, current smoker (smokes about two to three packs per day) who was brought in by the family to our emergency department with complaints of fever and altered mental status. As per the family, the night before the presentation she had gone to bed complaining of headache, and right-sided ear pain. In the morning she was found to be altered, febrile, and nonverbal. They reported no trauma, no recent domestic or international travel, no rashes, no recent camping trips, and no pets at home. A quick review of her chart revealed that the patient had come to our emergency department multiple times in the two weeks preceding her current visit. She had first presented to the Emergency Department (ED) 11 days prior with complaints of bilateral frontal headache for five days. She did not have any meningeal signs on examination, however, she has right-sided ear effusion with a bulging tympanic membrane. Labs in this visit were significant for WBC 22,000 (4800-10800 K/µL), and C-reactive protein (CRP) 20.45 (<0.3 mg/dL). A CT head and CT angiogram of the head and neck were negative for any acute findings (Figure 1 and Figure 2).

Her symptoms improved in the ED with symptomatic management and given no evident source for the leukocytosis and absence of fever she was discharged from the ED on augmentin with a close outpatient appointment for follow-up.

Her vitals on readmission revealed a temperature of 104 degrees F, pulse of 155 beats per minute, blood pressure of 159/96 mm Hg, and saturation of 96% on room air. Moreover, her urinalysis showed: ketones, large glucose, rare bacteria, rare WBC, nitrite, and leukocyte esterase negative. Urine toxicology was negative. On examination, she was altered, nonverbal, unable to follow commands, febrile, neck rigidity present, pupils equal and reactive, ears bilaterally unremarkable, chest and abdomen examination were normal and no rashes were seen. Her labs at the presentation are detailed in Table 1.

**Table 1:** Initial laboratory investigations

Investigation	Result	Normal Range
Hemoglobin (g/dl)	12.6	12.0-16.0
WBC (/uL)	23500	4800-10800
Platelets (/uL)	181000	150000-400000
Sodium (mEq/L)	129	135-145
Potassium (mEq/L)	4.2	3.5-5.0
Calcium (mEq/L)	8.7	8.5-10.5
Chloride (mEq/L)	99	98-108
Glucose (mg/dl)	359	70-120
Bicarbonate (mEq/L)	15	24-30
Blood Urea Nitrogen (mg/dl)	15	6-20
Creatinine (mg/dl)	1.0	0.5-1.5

Her hepatic function tests and coagulation markers were within normal limits. Her hemoglobin A1c was elevated at 11.3% (4.7-6.4%). Chest X-ray revealed multiple left lower lobe opacities. CT scan of the chest also revealed minimal right lower lobe bronchopneumonia. CT scan of the brain without contrast revealed a nonspecific soft tissue right tympanomastoidectomy cavity. Her LP from the current admission revealed a white blood count of 250/mm^3^ with neutrophilic predominance, glucose 32 mg/dl (reference range: 40-70 mg/dL), protein 341 mg/dl (reference range: 15-45 mg/dL), CSF lactic acid 68 and gram stain was positive for many leukocytes. However, her LPs from the previous two emergency visits were negative, including gram stain and culture. She was admitted to Intensive Care Unit for further management of septic encephalopathy secondary to bacterial meningoencephalitis, pneumonia, and mild diabetic ketoacidosis.

Infectious disease was consulted. Pneumonia workup, including Mycoplasma pneumoniae and urine legionella antigen, were negative. Initial blood cultures were positive for Klebsiella pneumonia, however, chest X-ray was negative for any cardiopulmonary disease. HIV antibodies were also negative. Magnetic resonance imaging (MRI) of the head was negative for cerebral venous thrombosis. Given high suspicion for sepsis due to meningitis/encephalitis she was immediately started empirically on intravenous dexamethasone 10 mg every 6 hours, ampicillin 2 g every 4 hours, ceftriaxone 2 g every 12 hours, vancomycin, and acyclovir 10 mg/kg every 8 hours. Because of her history of right ear pain, previous recent ED visits for the same complaints IV Metronidazole was added. The repeat blood cultures were negative. Later, acyclovir was stopped after CSF PCR for herpes simplex virus (HSV) was negative. Subsequently, antibiotics were de-escalated to vancomycin and ceftriaxone after completion of steroid treatment and improvement in symptoms, particularly mental status.

She completed 14 days of intravenous vancomycin and ceftriaxone and was discharged on augmentin 875-125 mg every 12 hours to complete 14 days given the right ear opacification. She was discharged with outpatient infectious disease and ENT follow-up and is improving well.

## Discussion

Bacterial meningitis is a life-threatening infection that needs immediate treatment. The prevalence of bacterial meningitis has exceeded 16 million cases, with mortality rates as high as 30% [2]. The mortality rates also depend upon the underlying microorganism responsible for bacterial meningitis and can vary, for example, 57% in meningococcal sepsis, 30% in pneumococcal, and 7% in meningococcal meningitis without sepsis [4]. Early identification is essential in reducing both incidence and mortality secondary to bacterial meningitis [5].

The clinical presentation of meningitis includes a triad of fever, headaches, and an altered mental status. However, half of the patients do not develop typical symptoms, therefore, the diagnosis of meningitis cannot be excluded. Bacterial meningitis with an initial normal analysis of normal CSF is rare. A small case series was done by Onorato et al. including five patients with normal initial CSF analysis, diagnosed bacterial meningitis upon a second lumbar puncture performed 8-36 hours later [6]. Lab investigations are also significant for elevated proteins, elevated WBCs with neutrophilic or lymphocytic predominance, low glucose levels, and varying opening pressure [7].

Before starting antibiotics, it is advisable to begin early treatment along with a series of blood cultures and cerebrospinal fluid (CSF) analysis via lumbar puncture (LP). Dexamethasone should be initiated in patients with bacterial meningitis before or with the initiation of antibiotics, however, its impact on mortality has not been studied yet [7]. Steroids help in the improvement of cerebral blood flow and therefore help in the prevention of Central Nervous System delayed complications and reducing intracranial pressure.

There is limited literature on adult patients with bacterial meningitis regarding the repetition of lumbar puncture [8]. Previously repeat lumbar puncture was recommended to determine the length of antimicrobial therapy based on normalization of CSF leukocyte counts, CSF glucose concentrations level, and CSF protein levels, however, additional recommendations include lack of clinical response after 48 hours of appropriate antimicrobial therapy, reducing intracranial pressure with communicating hydrocephalus, or in those who deteriorate after initial recovery [9-13]. Although initial lumbar puncture results, specifically cultures and gram stain, might be inconclusive if patients have been partially treated with antibiotics [14]. Our patient presented multiple times in ED due to worsening of her symptoms and due to her unusual presentation, she underwent LP twice.

In a retrospective study conducted on a similar cohort of patients, repeat lumbar puncture was needed in patients with suspicion of bacterial meningitis (79.0%), as observed in our case [14]. The procedure was commonly repeated in the first week of admission [14]. In our case, the patient had a similar clinical course and timeline during which the procedure was performed again. Repeated LPs were usually significant for elevated leukocyte counts in CSF and for bacterial culture [14]. Our patient, despite having leukocytosis in CSF fluid, did not have a positive CSF culture. CSF lactic acid levels on repeat LP are usually predictive of cure in adult patients with bacterial meningitis [15], however, reference ranges need to be constructed for the measure to be implemented practically. Additional radiological investigations are warranted in patients with pneumococcal meningitis provided a higher risk of venous thrombosis [12].

Antibiotics are the primary treatment for bacterial meningitis. The choice of antibiotics depends on the suspected or identified bacteria. In many cases, broad-spectrum antibiotics are initially administered until the specific bacteria causing the infection are identified through laboratory tests. Once the bacteria are identified, the antibiotics may be adjusted to a more targeted therapy. Commonly used antibiotics for bacterial meningitis include third-generation cephalosporins (e.g., ceftriaxone or cefotaxime) along with vancomycin or ampicillin to cover additional bacteria. Acyclovir is commended if herpes encephalitis is suspected which includes the symptoms such as loss of consciousness, focal neurological deficits, seizures, and as evident on imaging [5]. Our patient was later presented with altered mental and seizure-like activity, therefore, was started on acyclovir along with antibiotics.

Complications of acute bacterial meningitis in adults include impaired consciousness and recurrent convulsions that are commonly encountered in patients with older age or altered mental status on presentation [1].

## Conclusions

In conclusion, we discussed a case of bacterial meningitis which was diagnosed on subsequent LP. Rapid detection through CSF analysis facilitated further lesion detection leading to a full recovery of the patient. Our case highlights the importance of repeat LP as performing repeat lumbar puncture (LP) in cases where the initial LP results are inconclusive is essential for averting mortality and detecting potentially severe complications associated with meningitis.

## References

[1] GBD 2016 Meningitis Collaborators (2018). Global, regional, and national burden of meningitis, 1990-2016: a systematic analysis for the Global Burden of Disease Study 2016. Lancet Neurol.

[2] Niemelä S, Lempinen L, Löyttyniemi E, Oksi J, Jero J (2023). Bacterial meningitis in adults: a retrospective study among 148 patients in an 8-year period in a university hospital, Finland. BMC Infect Dis.

[3] McGill F, Heyderman RS, Panagiotou S, Tunkel AR, Solomon T (2016). Acute bacterial meningitis in adults. Lancet.

[4] Smith L (2005). Management of bacterial meningitis: new guidelines from the IDSA. Am Fam Physician.

[5] Moradi G, Zahraei SM, Khazaei Z (2021). Epidemiology incidence and geographical distribution of Meningitis using GIS and its incidence prediction in Iran in 2021. Med J Islam Repub Iran.

[6] Onorato IM, Wormser GP, Nicholas P (1980). 'Normal' CSF in bacterial meningitis. JAMA.

[7] Griffiths MJ, McGill F, Solomon T (2018). Management of acute meningitis. Clin Med (Lond).

[8] van de Beek D, de Gans J, Spanjaard L, Weisfelt M, Reitsma JB, Vermeulen M (2004). Clinical features and prognostic factors in adults with bacterial meningitis. N Engl J Med.

[9] Denneman L, Vial-Dupuy A, Gault N, Wolff M, van de Beek D, Mourvillier B (2013). Repeated lumbar puncture in adults with pneumococcal meningitis: an observational study. J Infect.

[10] Tunkel AR, Hartman BJ, Kaplan SL, Kaufman BA, Roos KL, Scheld WM, Whitley RJ (2004). Practice guidelines for the management of bacterial meningitis. Clin Infect Dis.

[11] Kasanmoentalib ES, Brouwer MC, van der Ende A, van de Beek D (2010). Hydrocephalus in adults with community-acquired bacterial meningitis. Neurology.

[12] Schut ES, Brouwer MC, de Gans J, Florquin S, Troost D, van de Beek D (2009). Delayed cerebral thrombosis after initial good recovery from pneumococcal meningitis. Neurology.

[13] Hoffman O, Weber RJ (2009). Pathophysiology and treatment of bacterial meningitis. Ther Adv Neurol Disord.

[14] Costerus JM, Brouwer MC, van der Ende A, van de Beek D (2016). Repeat lumbar puncture in adults with bacterial meningitis. Clin Microbiol Infect.

[15] Cunha BA (2013). Repeat lumbar puncture: CSF lactic acid levels are predictive of cure with acute bacterial meningitis. J Clin Med.

